# Intra-abdominal pressure and perfusion pressure in acute decompensated heart failure: clinical and prognostic insights

**DOI:** 10.1007/s11845-026-04371-6

**Published:** 2026-04-13

**Authors:** Selda Murat, Fatih Enes Durmaz, Ahmet Sekip Ahmadi, Zeynep Yuzuak, Bektas Murat, Yuksel Cavusoglu

**Affiliations:** 1 Medical Faculty Department of Cardiology, Eskisehir Osmangazi University, Eskisehir, Turkey; 2 Ministry of Health Mus State Hospital, Mus, Turkey; 3 Department of Cardiology, Eskisehir City Hospital, Eskisehir, Turkey

**Keywords:** Acute decompensated heart failure, Intra-abdominal pressure, Abdominal perfusion pressure, Abdominal congestion, Prognosis, Rehospitalization

## Abstract

**Background:**

Abdominal venous congestion is increasingly recognized as an important contributor to clinical deterioration in acute decompensated heart failure (ADHF). Intra-abdominal pressure (IAP) and abdominal perfusion pressure (APP) may provide complementary insights into systemic congestion and organ perfusion; however, their prognostic value in ADHF remains undefined.

**Aim:**

To evaluate the prognostic value of IAP and APP in patients with acute decompensated heart failure.

**Methods:**

This prospective observational study enrolled 110 consecutive patients hospitalized with ADHF. IAP was measured using a standardized transvesical technique within the first 24 h of admission, and intra-abdominal hypertension (IAH) was defined as an IAP ≥ 12 mmHg. APP was calculated as mean arterial pressure minus IAP. Clinical, echocardiographic, and laboratory characteristics were recorded. Mortality and rehospitalization at 1, 6, and 12 months were assessed, and multivariable regression analyses were performed to identify independent predictors of adverse outcomes.

**Results:**

IAH was present in 29.1% of patients. Compared with patients without IAH, those with IAH had lower diastolic and mean arterial pressures, larger right atrial dimensions, and worse renal function. Elevated IAP was associated with significantly higher mortality at 1, 6, and 12 months, as well as increased rehospitalization rates during follow-up. In multivariable analyses, elevated IAP remained an independent predictor of mortality and rehospitalization, whereas higher APP was associated with more favorable early outcomes.

**Conclusion:**

Elevated IAP is an independent predictor of adverse outcomes in ADHF, while APP provides complementary prognostic information. Integrating both congestion- and perfusion-related parameters may improve risk stratification and guide targeted decongestive strategies.

## Introduction

Acute decompensated heart failure (ADHF) remains a major cause of hospitalization and mortality worldwide, with systemic congestion being the central pathophysiological mechanism underlying clinical deterioration [1, 2]. Although relief of congestion is the primary therapeutic goal, conventional assessment based on physical examination and changes in body weight is often subjective and insufficient [1, 3, 4]. Consequently, a substantial proportion of patients are discharged with residual congestion, which has been consistently associated with higher rates of rehospitalization and long-term mortality [5, 6]. This underscores the need for more objective and reproducible parameters to evaluate congestion and guide therapy in ADHF.

Recent research has highlighted the value of biomarkers such as natriuretic peptides, as well as imaging modalities including lung ultrasound and inferior vena cava assessment, for improving congestion evaluation [7, 8]. However, these methods may not fully capture the hemodynamic burden in certain patients, particularly in the setting of systemic venous congestion. In this context, intra-abdominal pressure (IAP) has emerged as a potential marker of abdominal venous congestion and elevated central venous pressure. Elevated IAP has been associated with worsening renal function, impaired diuretic responsiveness, and adverse clinical outcomes in patients with ADHF [9–13]. Abdominal perfusion pressure (APP), defined as the difference between mean arterial pressure and IAP, further refines the assessment of effective organ perfusion by integrating both perfusion pressure and venous congestion [14]. As such, APP may provide incremental prognostic information beyond IAP alone, especially in patients with compromised hemodynamics [14].

Despite this pathophysiological rationale, the clinical role of IAP and APP in patients with ADHF has not been clearly established. Most of the existing evidence derives from small studies or experimental models focusing primarily on reduced ejection fraction, while the prognostic relevance across a broader spectrum of heart failure phenotypes remains uncertain [9]. Moreover, the potential of IAP and APP to predict long-term mortality and rehospitalization has not been systematically addressed.

In this study, we aimed to investigate the relationship between IAP, APP, and clinical outcomes in patients admitted with ADHF. Specifically, we assessed their relationships with markers of congestion, renal function, length of hospital stay, and long-term mortality. By addressing these gaps, our study seeks to provide new insights into the prognostic utility of IAP and APP in the management of ADHF.

## Methods

### Study population

This study was conducted in accordance with the Declaration of Helsinki and was approved by the Eskisehir Osmangazi University Non-Interventional Clinical Trials Ethics Committee (Approval Number: 23, Date: 15.02.2022). This prospective observational study was conducted between March 30, 2022, and March 30, 2024, at Eskişehir Osmangazi University Faculty of Medicine. A total of 110 consecutivepatients admitted with a diagnosis of ADHF were enrolled in the study. Written informed consent was obtained from all patients.

The inclusion criteria comprised patients aged 18 years or older who were admitted with a diagnosis of ADHF and provided informed consent for participation. Exclusion criteria were as follows: presence of urinary tract infection, intra-abdominal mass, need for mechanical ventilation, history of abdominal or thoracic surgery within the last six months, absence of a Foley catheter, or ongoing renal replacement therapy.

All relevant clinical information, including demographic data, comorbidities, medication history, echocardiographic data, and laboratory findings, was prospectively collected at the time of hospital admission.

### Intra-abdominal pressure and abdominal perfusion pressure measurement

IAP was measured prospectively using the standardized transvesical technique, in accordance with previously published recommendations [9, 14, 15]. The measurement procedure was as follows: a standard Foley catheter was inserted, and the drainage tube was clamped. Through the aspiration port, a maximum of 25 mL sterile saline was instilled to establish a fluid-filled column within the bladder. Care was taken to avoid excessive instillation, which may cause bladder distension, patient discomfort, and falsely elevated pressure readings. The catheter was connected to a pressure transducer zeroed at the level of the iliac crest along the midaxillary line. IAP was recorded in mmHg at end-expiration with the patient in the supine position, ensuring the absence of abdominal muscle contractions. IAP measurements were performed at two time points: within the first 24 h of hospital admission and immediately prior to discharge, at the time the treating physician deemed the patient clinically stable for discharge.

In this study, we referenced the most recent consensus definitions from the World Society of the Abdominal Compartment Syndrome (WSACS, 2013) [16]. According to these guidelines, normal IAP in critically ill adults is approximately 5–7 mmHg, while intra-abdominal hypertension (IAH) is defined as a sustained or repeated IAP ≥ 12 mmHg. Therefore, for the purposes of this study, we designated IAP ≥ 12 mmHg as the threshold for IAH, and used this criterion to classify patients accordingly. APP was calculated as the difference between mean arterial pressure (MAP) and IAP (APP = MAP – IAP). Comparisons were performed between patients with and without adverse outcomes to evaluate differences in clinical characteristics, laboratory findings, and hemodynamic parameters, including IAP, APP, and MAP.

### Follow-up and outcomes

All patients were prospectively followed at two predefined time points: 6 months and 1 year after the index hospitalization. The composite outcome was defined as 1-year rehospitalization due to heart failure and all-cause mortality. Clinical characteristics, laboratory findings, and hemodynamic parameters including IAP, APP, and MAP were compared between patients who experienced adverse outcomes and those who did not.

### Statistical analysis

Statistical analyses were performed using SPSS Statistics, version 23.0 (IBM Corp., Armonk, NY, USA). Continuous variables were assessed for normality using the Shapiro–Wilk test and are presented as mean ± standard deviation or median (interquartile range) as appropriate. Categorical variables were summarized as frequencies and percentages. Group comparisons between patients with IAH (IAP ≥ 12 mmHg) and those with lower IAP values were conducted using the independent samples t-test or Mann–Whitney U test for continuous variables and the chi-square test or Fisher’s exact test for categorical variables, as appropriate.

To evaluate the association between clinical, laboratory, and hemodynamic parameters and adverse outcomes, univariate and multivariable logistic regression analyses were performed for each predefined clinical endpoint, including 1-month, 6-month, and 12-month mortality; 1-month, 6-month, and 12-month rehospitalization; and the composite outcome. For each endpoint, univariate logistic regression analyses were initially conducted using candidate variables selected a priori based on clinical relevance and prior literature. These variables included age, sex (male vs. female), systolic blood pressure at admission, serum sodium and potassium levels, N-terminal pro–B-type natriuretic peptide (NT-proBNP), maximum creatinine, systolic pulmonary artery pressure (SPAP), MAP, IAP, APP, and left ventricular ejection fraction.

Variables with a p value < 0.10 in the univariate analyses for a given endpoint were subsequently entered into the corresponding multivariable logistic regression model. Because the set of significant univariate predictors differed across endpoints, each multivariable model incorporated a distinct combination of covariates derived solely from its own univariate screening. Results of the multivariable analyses are reported as odds ratios (ORs) with 95% confidence intervals (CIs), and only variables that remained independently associated with outcomes after adjustment are presented in Table 4.

## Result

A total of 110 patients hospitalized with ADHF were included in the analysis, of whom 32 (29.1%) had IAH (IAP ≥ 12 mmHg). Age and sex distribution were comparable between groups, whereas diabetes mellitus was significantly more prevalent in patients with elevated-IAP group (65.6% vs. 35.9%, *p* = 0.004). At intensive care unit (ICU) admission, patients in the elevated-IAP group presented with lower diastolic and mean blood pressures (DBP: 80 vs. 71 mmHg, *p* = 0.008; MAP: 100 vs. 96.6 mmHg, *p* = 0.021). Chronic comorbidities such as hypertension and coronary artery disease were similar between groups.

Regarding medications, the use of angiotensin-converting enzyme inhibitor, angiotensin II receptor blocker, angiotensin receptor–neprilysin inhibitor, beta-blockers, mineralocorticoid receptor antagonists, sodium–glucose cotransporter-2 inhibitors, and loop diuretics did not differ significantly between the groups. As expected, mean IAP measured during the first 24 h was significantly higher in the elevated-IAP group (14.2 ± 2.6 vs. 7.9 ± 2.1 mmHg, *p* < 0.001). Although APP tended to be lower in patients with IAP ≥ 12 mmHg, the difference did not reach statistical significance (74.5 ± 14.2 vs. 79.5 ± 12.9 mmHg, *p* = 0.076). MAP values were also comparable between groups. These findings are summarized in detail in Table 1, which presents the comparative clinical, hemodynamic, and laboratory characteristics of patients stratified by IAP levels.

**Table 1:** Baseline clinical characteristics according to intra-abdominal pressure status

Characteristics	IAP ≥ 12 mmHg (*n* = 32)	IAP < 12 mmHg (*n* = 78)	*p* value
Age, years	74.5 ± 9.1	71.8 ± 7.4	0.147
Female Sex, n (%)	19 (59.4%)	38 (48.7%)	0.210
SBP at ED admission, mmHg	127.0 (108.2–177)	155.0 (134.7–170)	0.710
DBP at ED admission, mmHg	80.0 (62.0-103.2)	83.5 (75.0-98.5)	0.796
MAP at ED admission, mmHg	93.6 (79.4–124.0)	106.3 (94.3–125.0)	0.227
Heart rate at ED admission, bpm	100.5 (74.5–117.0)	100.0 (85.0-111.5)	0.443
SBP at ICU admission, mmHg	138.0 (122.0-157.7)	133.0 (120.0-156.5)	0.761
DBP at ICU admission, mmHg	80.0 (74.0-88.5)	71.0 (65.0–80.0)	0.008
MAP at ICU admission, mmHg	100.0 (93.3-106.9)	96.6 (86.6-103.6)	0.021
Heart rate at ICU admission, bpm	89.0 (78.2–99.7)	83.0 (75.0–98.0)	0.933
Hypertension, n(%)	22 (68.8%)	51 (65.4%)	0.458
Diabetes, n(%)	21 (65.6%)	28 (35.9%)	0.004
CAD, n(%)	17 (53.1%)	52 (66.7%)	0.132
CKD, n(%)	8 (24.4%)	19 (25.0%)	0.562
ACE-I/ARB/ARNI, n(%)	24 (75.0%)	61 (78.2%)	0.447
Beta-blockers, n(%)	23 (71.9%)	65 (83.3%)	0.136
MRA, n(%)	12 (37.5%)	27 (34.6%)	0.469
SGLT-2 inhibitor, n(%)	11 (34.4%)	29 (37.2%)	0.480
Diuretics, n(%)	27 (84.4%)	64 (82.1%)	0.504
IAP at first 24 h, mmHg	14.2 ± 2.6	7.9 ± 2.1	< 0.001
APP at first 24 h, mmHg	74.5 ± 14.2	79.5 ± 12.9	0.076
MAP at first 24 h, mmHg	88.8 ± 13.2	87.6 ± 12.7	0.668

*Continuous variables are presented as mean ± SD or median (interquartile range), as appropriate, and categorical variables are presented as number (percentage)

Abbreviations: *ACE-I,* Angiotensin-converting enzyme inhibitor; *ARB,* Angiotensin II receptor blocker; *ARNI,* Angiotensin receptor–neprilysin inhibitor; *APP,* Abdominal perfusion pressure; *CAD,* Coronary artery disease; *CKD,* Chronic kidney disease; *ED,* Emergency department; *IAP,* Intra-abdominal pressure; *ICU,* Intensive care unit; *MAP,* Mean arterial pressure; *MRA,* Mineralocorticoid receptor antagonist; *SGLT-2 inhibitor,* Sodium–glucose cotransporter-2 inhibitor

Echocardiographic measurements, including left ventricular ejection fraction (LVEF) (41.2 ± 14.5% vs. 39.9 ± 15.4%, *p* = 0.690), LV dimensions, interventricular septal thickness, mitral E velocity, and SPAP (50.5 [31.2–71.7] mmHg vs. 37.0 [30.0–52.0] mmHg, *p* = 0.079), were similar between the groups (all *p* > 0.05). However, right atrial dimension was significantly larger in the elevated-IAP group (46.8 ± 10.7 mm vs. 42.5 ± 8.0 mm, *p* = 0.027).

In terms of laboratory findings, patients with IAP ≥ 12 mmHg exhibited significantly lower serum sodium levels (131.0 ± 3.6 mEq/L vs. 133.1 ± 4.1 mEq/L, *p* = 0.022) and lower potassium levels (4.37 ± 0.56 mEq/L vs. 4.61 ± 0.49 mEq/L, *p* = 0.036). While baseline creatinine showed only a trend toward higher values (1.65 [1.25–1.82] mg/dL vs. 1.23 [1.01–1.36] mg/dL, *p* = 0.070), markers of renal deterioration were clearly worse in this group: creatinine at discharge (1.70 [1.18–2.84] mg/dL vs. 1.28 [0.99–1.61] mg/dL, *p* = 0.003) and maximum creatinine during hospitalization (2.21 [1.37–3.06] mg/dL vs. 1.36 [1.06–2.17] mg/dL, *p* = 0.011) were significantly elevated compared with patients with IAP < 12 mmHg. NT-proBNP values were comparable between the groups (*p* = 0.519). Echocardiographic and laboratory comparisons are summarized in Table 2.

**Table 2:** Echocardiographic, and laboratory characteristics according to IAP status

Characteristics	IAP ≥ 12 mmHg (*n* = 32)	IAP < 12 mmHg (*n* = 78)	*p* value*
LVEF, %	41.2 ± 14.5	39.9 ± 15.4	0.690
LV end-diastolic diameter, mm	52.9 ± 9.1	53.7 ± 8.9	0.667
LV end-systolic diameter, mm	39.8 ± 11.9	40.3 ± 10.9	0.688
IVS, mm	11.5 ± 1.6	11.2 ± 1.9	0.452
Mitral E velocity, cm/s	91.0 (68.0-102.5)	82.0 (49.5–98.0)	0.732
Left atrium dimension, mm	43.7 ± 5.0	45.4 ± 6.3	0.189
Right atrium dimension, mm	46.8 ± 10.7	42.5 ± 8.0	0.027
Tricuspid regurgitation velocity, m/s	3.0 ± 0.85	2.7 ± 0.74	0.152
SPAP, mmHg	50.5 (31.2–71.7)	37.0 (30.0–52.0)	0.079
Sodium, mEq/L	131.0 ± 3.6	133.1 ± 4.1	0.022
Potassium, mEq/L	4.37 ± 0.56	4.61 ± 0.49	0.036
NT-proBNP, pg/mL	6202.0 (1904.0-13197.0)	5478.0 (2579.2-33186.0)	0.519
Creatinine, mg/dL	1.65 (1.25–1.82)	1.23 (1.01–1.36)	0.070
Creatinine at discharge, mg/dL	1.70 (1.18–2.84)	1.28 (0.99–1.61)	0.003
Maximum creatinine, mg/dL	2.21 (1.37–3.06)	1.36 (1.06–2.17)	0.011

* Continuous variables are presented as mean ± standard deviation or median (interquartile range), as appropriate. Continuous variables were compared using the independent samples t-test or Mann–Whitney U test, and categorical variables were compared using the chi-square or Fisher’s exact test. A p-value < 0.05 was considered statistically significant

Abbreviations: *IAP,* Intra-abdominal pressure; *IVS,* Interventricular septum; *LVEF,* Left ventricular; *NT-proBNP,* N-terminal pro–B-type Natriuretic peptide; *SPAP,* Systolic pulmonary artery pressure

### Follow-up outcomes

During follow-up, elevated IAP (IAP ≥ 12 mmHg) was associated with worse clinical outcomes compared with lower IAP values (Table 3). One-month mortality was higher in the elevated-IAP group (15.6% vs. 3.8%, *p* = 0.045), and this difference further increased at six months (31.3% vs. 10.3%, *p* = 0.010) and one year (43.8% vs. 16.7%, *p* = 0.004). Similarly, rehospitalization rates at one and six months were significantly higher in patients with IAP ≥ 12 mmHg (34.4% vs. 14.1%, *p* = 0.018; and 43.8% vs. 20.5%, *p* = 0.015, respectively), whereas no significant difference was observed at one year (*p* = 0.480).

**Table 3:** Short- and long-term clinical outcomes according to IAP category

Characteristics	IAP ≥ 12 mmHg (*n* = 32)	IAP < 12 mmHg (*n* = 78)	*p* value*
In-hospital mortality, n(%)	3 (9.4%)	3 (3.8%)	0.234
1-month mortality, n(%)	5 (15.6%)	3 (3.8%)	0.045
6-month mortality, n(%)	10 (31.3%)	8 (10.3%)	0.010
1-year mortality, n(%)	14 (43.8%)	13 (16.7%)	0.004
1-month rehospitalization, n(%)	11 (34.4%)	11 (14.1%)	0.018
6-month rehospitalization, n(%)	14 (43.8%)	16 (20.5%)	0.015
1-year rehospitalization, n(%)	11 (34.4%)	29 (37.2%)	0.480
Length of hospital stay, days	7.5 (4.2–13)	5 (3–7)	0.012
Composite outcome, n(%)	22 (68.8%)	34 (43.6%)	0.014

*Categorical variables are presented as n (%) and compared using Fisher’s exact test. Continuous variables are presented as median (interquartile range) and compared using the Mann–Whitney U test. A *p*-value < 0.05 was considered statistically significant. IAP: Intra-abdominal pressure

Patients with elevated IAP had a longer length of hospital stay (median 7.5 [4.2–13] vs. 5 [3–7] days, *p* = 0.012). The composite endpoint, consisting of 1-year heart failure rehospitalization and all-cause mortality, was significantly higher in the IAP ≥ 12 mmHg group (68.8% vs. 43.6%, *p* = 0.014). In-hospital mortality was numerically higher in patients with elevated IAP, although the difference did not reach statistical significance (9.4% vs. 3.8%, *p* = 0.234).

### Predictors of adverse outcomes

To identify variables independently associated with adverse outcomes, separate univariate and multivariable logistic regression models were constructed for each predefined endpoint, including 1-month, 6-month, and 12-month mortality; 1-month, 6-month, and 12-month rehospitalization; and the composite outcome.

For each endpoint, univariate logistic regression analyses were initially performed using the following candidate variables: age, sex (male vs. female), SBP at admission, serum sodium and potassium levels, NT-proBNP, maximum creatinine, SPAP, MAP, IAP, APP, LVEF.

Variables with *p* < 0.10 in the univariate analyses for a given endpoint were subsequently entered into the corresponding multivariable logistic regression model. As the set of significant univariate predictors differed across endpoints, each multivariable model included a distinct combination of covariates derived exclusively from its respective univariate screening.

**Table 4:** Multivariable logistic regression analysis of predictors of mortality and rehospitalization across different time points

Outcomes	Variables	β	Standard Error (SE)	Wald z statistic	p	Odds Ratio (OR)	95% CI Lower-Upper Bound
1-month mortality	APP	-0.134	0.047	-2.854	0.004	0.88	0.80-0.96
	LVEF	0.068	0.038	1.793	0.073	1.07	0.99-1.15
6-month mortality	IAP	0.168	0.075	2.247	0.024	1.18	1.02-1.37
	MAP	-0.087	0.027	-3.234	0.001	0.92	0.87-0.97
1-year mortality	SPAP	0.035	0.016	2.165	0.030	1.04	1.00-1.07
	IAP	0.261	0.088	2.954	0.003	1.30	1.09-1.54
	MAP	-0.092	0.027	-3.441	0.001	0.91	0.87-0.96
1-month rehospitalization	Age	-0.074	0.029	-2.553	0.010	0.93	0.88-0.98
	APP	-0.042	0.020	-2.086	0.037	0.96	0.92-1.00
6-month rehospitalization	Potassium	-0.939	0.479	-1.959	0.050	0.39	0.15-1.00
	APP	-0.055	0.019	-2.883	0.003	0.95	0.91-0.98
	Sex (male)	-1.242	0.499	-2.488	0.012	0.29	0.11-0.77
1-year rehospitalization	Age	-0.055	0.025	-2.232	0.025	0.95	0.90-0.99
	SBP	-0.027	0.012	-2.268	0.023	0.97	0.95-1.00
Composite outcome	IAP	0.216	0.070	3.106	0.001	1.24	1.08-1.42
	MAP	-0.092	0.021	-4.301	0.001	0.91	0.88-0.95

Wald z statistics were calculated as β divided by the standard error

Abbreviations: *APP,* Abdominal perfusion pressure; *IAP,* Intra-abdominal pressure; *LVEF,* Left ventricular ejection fraction; *MAP,* Mean arterial pressure; *SPAP,* Systolic pulmonary artery pressure; *SBP,* Systolic blood pressure

### Rehospitalization outcomes (1-, 6-, and 12-month)

According to univariate logistic regression analyses, variables associated with rehospitalization differed across follow-up periods. For 1-month rehospitalization, age (OR 1.05, 95% CI 1.01–1.09, *p* = 0.012), IAP measured at admission (OR 1.18, 95% CI 1.08–1.28, *p* < 0.001), and APP (OR 0.95, 95% CI 0.91–0.99, *p* = 0.021) were significantly associated with rehospitalization.

For 6-month rehospitalization, IAP (OR 1.14, 95% CI 1.06–1.23, *p* = 0.001) and APP (OR 0.94, 95% CI 0.90–0.98, *p* = 0.006) remained significantly associated with rehospitalization, whereas no other clinical or hemodynamic variables reached statistical significance.

For 12-month rehospitalization, univariate analyses identified age (OR 1.06, 95% CI 1.02–1.10, *p* = 0.004), SBP at admission (OR 0.97, 95% CI 0.95–0.99, *p* = 0.015), MAP (OR 0.96, 95% CI 0.93–0.99, *p* = 0.022), and APP (OR 0.93, 95% CI 0.89–0.97, *p* = 0.003) as significant correlates of rehospitalization.

### Mortality and composite outcomes (1-, 6-, and 12-month)

Univariate logistic regression analyses demonstrated that variables associated with mortality differed across follow-up intervals. For 1-month mortality, age (OR 1.06, 95% CI 1.01–1.11, *p* = 0.019), diabetes mellitus (OR 3.24, 95% CI 1.07–9.78, *p* = 0.038), IAP (OR 1.19, 95% CI 1.05–1.35, *p* = 0.006), APP (OR 0.94, 95% CI 0.88–0.99, *p* = 0.028), and creatinine at discharge (OR 2.20, 95% CI 1.26–3.86, *p* = 0.006) were significantly associated with mortality.

For 6-month mortality, age (OR 1.05, 95% CI 1.01–1.09, *p* = 0.015), diabetes mellitus (OR 2.73, 95% CI 1.14–6.52, *p* = 0.024), IAP (OR 1.13, 95% CI 1.04–1.22, *p* = 0.003), serum potassium level (OR 2.11, 95% CI 1.03–4.31, *p* = 0.040), as well as both admission and discharge creatinine levels (OR 3.35, 95% CI 1.67–6.72, *p* = 0.001 and OR 2.45, 95% CI 1.45–4.12, *p* = 0.001, respectively) were significantly associated with mortality.

For 12-month mortality, age (OR 1.05, 95% CI 1.02–1.09, *p* = 0.003), diabetes mellitus (OR 2.32, 95% CI 1.08–4.96, *p* = 0.031), serum potassium level (OR 2.21, 95% CI 1.15–4.25, *p* = 0.018), IAP (OR 1.12, 95% CI 1.04–1.20, *p* = 0.003), and maximum creatinine level during hospitalization (OR 1.42, 95% CI 1.10–1.84, *p* = 0.007) were significantly associated with mortality.

In univariate logistic regression analyses, several variables were significantly associated with the composite endpoint. Age (OR 1.04, 95% CI 1.01–1.07, *p* = 0.009) and serum potassium level (OR 2.05, 95% CI 1.11–3.78, *p* = 0.021) were significantly associated with an increased risk of the composite outcome. Markers of renal dysfunction, including creatinine at discharge (OR 1.76, 95% CI 1.19–2.61, *p* = 0.005) and maximum creatinine during hospitalization (OR 1.27, 95% CI 1.06–1.53, *p* = 0.010), were also significantly associated with adverse outcomes.

Elevated IAP was strongly associated with an increased risk of the composite outcome (OR 1.12, 95% CI 1.05–1.20, *p* < 0.001), whereas APP demonstrated a favourable association (OR 0.94, 95% CI 0.91–0.98, *p* = 0.003).

Multivariable logistic regression models were constructed separately for each clinical endpoint using variables that demonstrated statistical significance in the corresponding univariate analyses (*p* < 0.10). Because the set of significant predictors differed across outcomes, each final multivariable model included a distinct combination of covariates derived from its respective univariate screening. Only variables that remained independently associated with outcomes after adjustment are presented in Table 4. Notably, elevated IAP remained an independent predictor of adverse outcomes, whereas higher APP consistently exhibited a favourable prognostic association.

## Discussion

In this prospective study of patients hospitalized with ADHF, we demonstrate that elevated IAP measured within the first 24 h of admission is a strong and independent predictor of adverse clinical outcomes, including early, intermediate, and long-term mortality and rehospitalization. The prognostic impact of IAP persisted after adjustment for age, renal function, diabetes, potassium levels, blood pressure parameters, and echocardiographic measurements, underscoring its value as an objective marker of systemic venous congestion. In contrast, APP showed a favourable prognostic association, particularly for short-term outcomes. These findings emphasize the contribution of abdominal congestion and impaired abdominal organ perfusion to clinical deterioration in ADHF and highlight their potential role in risk stratification.

The biological plausibility of these associations is well supported. Elevated IAP promotes renal venous congestion, reduces glomerular filtration, triggers neurohormonal activation, and diminishes diuretic responsiveness, mechanisms closely linked to adverse outcomes in heart failure [9–11, 17, 18]. Prior work by Mullens et al. first demonstrated that IAP measured via the transvesical technique was frequently elevated in ADHF and that reductions in IAP during decongestion paralleled improvements in renal function, suggesting a causal role in cardiorenal impairment rather than a passive reflection of fluid overload [9]. Moreover, evidence indicates that venous hypertension, rather than reduced forward flow, is the predominant driver of worsening renal function in this context, supporting the pathophysiologic importance of congestion-related hemodynamics [17].

Further insights into the role of IAP in ADHF have come from recent clinical studies. Rubio-Gracia et al. showed that patients with higher admission IAP had more pronounced renal dysfunction and impaired diuretic response, and that persistence of IAP ≥ 12 mmHg over 72 h predicted prolonged hospitalization and increased 1-year mortality [11]. Our findings parallel these observations, as patients with elevated IAP similarly exhibited worse renal parameters and poorer outcomes. Importantly, our study extends these findings by demonstrating that the prognostic impact of elevated IAP persists across multiple clinically relevant follow-up intervals, reflecting a sustained influence of abdominal congestion beyond the acute phase.

Additional contemporary evidence supports the link between elevated IAP, reduced abdominal perfusion, and diuretic resistance. A recent prospective study of 83 ADHF patients found that each 1-mmHg increase in IAP was associated with a marked reduction in 24- and 48-hour diuresis and lower APP [19]. This study reinforces the mechanistic link between elevated IAP, reduced perfusion, and diuretic resistance, and highlights the potential utility of IAP and APP as bedside tools for predicting early treatment response. Our findings extend this evidence by demonstrating that the clinical significance of elevated IAP is not restricted to early decongestion failure but also translates into adverse medium- and long-term outcomes, including mortality and rehospitalization.

The variability in reported prevalence of elevated IAP across studies likely reflects substantial differences in patient selection and baseline disease severity. The cohort examined by Łagosz et al. consisted of a small, highly selected group of patients with more advanced systemic congestion, greater hemodynamic compromise, and a markedly impaired diuretic response, clinical features that inherently predispose to higher IAP values and likely contributed to their elevated IAP prevalence of approximately 63.3% [12]. In contrast, our study enrolled a larger and more heterogeneous ADHF population representative of routine clinical practice, encompassing a broader range of systolic function, congestion severity, and comorbidity burden. This more diverse clinical profile likely explains the lower prevalence of elevated IAP in our cohort (29.1%). Notably, despite these differences, patients with elevated IAP in our study exhibited similarly impaired renal function and substantially worse clinical outcomes, underscoring the robustness of IAP as a prognostic marker across diverse heart failure phenotypes and severity levels.

Our study adds several important contributions to the literature. First, by evaluating mortality and rehospitalization at multiple time points, we demonstrate that IAP conveys prognostic information across early, intermediate, and late follow-up, an aspect not addressed in prior studies focused mainly on short-term renal or hemodynamic consequences. Second, we systematically evaluated APP alongside IAP, showing that APP provides additional prognostic information through its integration of perfusion and congestion physiology. Third, our findings strengthen the mechanistic link between abdominal congestion and kidney injury, as elevated IAP was associated with higher peak and discharge creatinine levels, indicating active cardiorenal dysfunction with downstream clinical consequences.

Taken together, these results support incorporating IAP and potentially APP into routine risk assessment in ADHF. Both measures are simple, reproducible, and capture physiologic domains that are not adequately reflected by conventional congestion markers. Early identification of patients with elevated IAP may enable more tailored decongestive strategies and improved monitoring, ultimately enhancing clinical outcomes.

### Limitations

This study has several limitations. First, it was conducted at a single center with a relatively limited sample size, which may limit the generalizability of the findings. Second, although IAP was measured using a standardized transvesical technique, measurements were obtained at only two predefined time points, within the first 24 h of admission and at discharge, and therefore may not fully capture dynamic fluctuations in IAP during hospitalization. Third, APP was derived from noninvasive blood pressure measurements, which may be subject to measurement variability. Fourth, although extensive multivariable adjustment was performed, residual confounding from unmeasured factors, including variability in diuretic strategies, splanchnic blood pooling, or venous compliance, cannot be excluded. Finally, the observational design precludes causal inference; thus, while elevated IAP and lower APP were strongly associated with adverse outcomes in this cohort, interventional studies are needed to determine whether targeted reduction of IAP can improve clinical outcomes in ADHF.

## Conclusion

In this prospective study of patients hospitalized with acute decompensated heart failure, elevated IAP was identified as a robust and independent predictor of adverse clinical outcomes, including mortality, rehospitalization, and a composite endpoint of major events across early, intermediate, and long-term follow-up. Conversely, APP demonstrated a favourable prognostic profile, suggesting that the interplay between congestion and perfusion carries important clinical implications beyond traditional markers of volume status. The strong associations between elevated IAP, impaired renal function, and adverse outcomes highlight the pathophysiological relevance of abdominal congestion as an active mediator of cardiorenal dysfunction rather than a passive indicator of fluid overload. Given the simplicity and reproducibility of transvesical IAP measurement, integrating IAP, and potentially APP, into routine risk stratification may help identify high-risk patients who could benefit from more intensive or targeted decongestive strategies. Future interventional studies are warranted to determine whether therapies aimed at reducing IAP and optimizing abdominal perfusion can translate into improved outcomes in ADHF.

## Data Availability

The datasets generated and/or analyzed during the current study are available from the corresponding author on reasonable request.
